# Resveratrol: brain effects on SIRT1, GPR50 and photoperiodic signaling

**DOI:** 10.3389/fnmol.2015.00061

**Published:** 2015-10-08

**Authors:** Joerg R. Leheste, German Torres

**Affiliations:** Department of Biomedical Sciences, NYIT College of Osteopathic MedicineOld Westbury, NY, USA

**Keywords:** SIRT1, GPR50, resveratrol, melatonin signaling, photoperiodic signaling, major depression

## Abstract

Silent information regulator-1 (SIRT1) deacetylase, a sensor of intermittent energy restriction, is inextricably intertwined with circadian regulation of central and peripheral clock genes. The purpose of this study was to identify SIRT1-specific target genes that are expressed in a circadian rhythm pattern and driven, in part, by specific components of foodstuffs. Using human cells and rats fed with a resveratrol diet we show that SIRT1 binds to, and transcriptionally regulates, a gene locus encoding the G protein-coupled receptor (GPR), GPR50 in the brain. GPR50 is the mammalian orthologue of the melatonin1c membrane-bound receptor which has been identified as a genetic risk factor for bipolar disorder and major depression in women. In general, our findings support and expand the notion that circadian clock signaling components and dietary interventions are adaptively linked, and suggest that the brain may be particularly sensitive to metabolic events in response to light-dark cycles.

## Introduction

Our physiology is driven by internal timing systems known as circadian clocks. Disruption of the clock contributes to the pathophysiology of several psychiatry disorders, including seasonal affective disorder, major depressive disorder, bipolar disorder and schizophrenia (Radeljak et al., 2008; Boyce and Barriball, 2010; Westrich and Sprouse, 2010). However, it is not clear whether disruption of rhythmic physiology *per se*, or altered functioning of individual clock components drives pathology. Nevertheless, the human silent information regulator-1 analog (SIRT1) appears to play a significant role in the clock-interdependent signaling mechanisms as SIRT1 modifies the feedback loop expression of circadian proteins, such as BMAL1 and PER2 (Asher et al., 2008; Nakahata et al., 2008, 2009; Ramsey et al., 2009). These findings suggest that rhythmic regulation of clock proteins is mediated by post-translational modifications driven, in part, by metabolic sensors such as SIRT1.

In this article, we report that the G protein-coupled receptor 50 (GPR50; Reppert et al., 1996) is a SIRT1-specific DNA target as demonstrated in human and rodent cells. GPR50 represents the mammalian orthologue of the vertebrate receptor MEL1c (Dufourny et al., 2008) and shares biochemical similarity with the two known melatonin receptors, MT1 and MT2. Despite structural similarities and the ability to dimerize with MT1 and MT2 receptors (Levoye et al., 2006), GPR50 does not bind melatonin and it is therefore classified as an orphan receptor with unknown endogenous or synthetic ligand(s). The hormone melatonin is secreted by the pineal gland and functions in the regulation of neural and endocrine processes, including readjusting the circadian pacemaker, the suprachiasmatic nucleus of the hypothalamus (SCN), to photo-period signals (Torres et al., 1989). To receive and relay melatonin photo-periodic transmission codes to appropriate neural networks, MT1 and MT2 must assemble into functional homo- or heterodimer (e.g., MT1/MT1; MT1/MT2; MT2/MT2) configurations within the bilayer lipid membrane to initiate second messenger cascades. For instance, whereas endogenous GPR50/MT2 heterodimers are melatonin responsive, MT1/GPR50 heterodimers are relatively silent to periodic depolarization pulses from pinealocytes (Levoye et al., 2006). The spatiotemporal diversity of these interactions highlights the wide range of GPR50-mediated responses to extracellular signaling events, which in turn converge on processes regulating gene transcription and the synthesis of proteins by local ribosomes.

To test the hypothesis that GPR50 is a DNA target directly affected by SIRT1, *in vivo* assays in conjunction with *in vitro* experiments were used to accomplish this experimental goal. More specifically, we used a previously described ChIP-cloning chromatin immunoprecipitation cell assay which links SIRT1 to transcriptional modification of certain endogenous DNA targets (Torres et al., 2008). For the *in vivo* experiments, we fed rats with trans-resveratrol (trans-3,5,4′-trihydroxystilbene) which is one of the signaling cues that activate SIRT1 transcription in the mammalian brain (Baur and Sinclair, 2006; Torres et al., 2011). Our results identify a new SIRT1 DNA target and highlight the significance of the trans-membrane GPR50 receptor in metazoan circadian rhythms and metabolic status.

## Materials and Methods

### Culture, Treatment, and Collection of Human Cells

Adherent human embryonic kidney cells (HEK-293; CRL-1573; ATCC, VA, USA) and a mixture of floating and adherent human neuroblastoma cells (SH-SY5Y; CRL-2266; ATCC, Manassas, VA, USA) were grown under standard culture conditions at 37°C in a humidified atmosphere with 5% CO_2_ on 75 mm^2^–100 mm^2^ or multiwell cell culture dishes (6–24 well). As base medium for HEK-293 cells we used Dulbecco’s Modified Eagle Medium (DMEM) whereas the base for SH-SY5Y was a 1:1 mixture of Eagle’s Minimum Essential Medium (EMEM) and F12 medium (Invitrogen/Life Technologies, Carlsbad, CA, USA). For induction and maintenance of a dopamine neuronal phenotype, SH-SY5Y cells were supplemented with 1 μM all trans-retinoic acid (Sigma-Aldrich, St. Louis, MO, USA) throughout the experiment. Immediately before use, supplements (Invitrogen/Life Technologies, Carlsbad, CA, USA) were added to the following reagents to yield their final concentrations of: 10% fetal bovine serum, 1%, penicillin/streptomycin, 1% non-essential amino acids, 1% stabile glutamine (Glutamax), and 1% anti-mycotic (Fungizone). At a culture density of approximately 70%, cells were subcultured 1:10 using 0.25% (w/v) Trypsin/0.53 mM EDTA. All cell treatments were initiated at a cellular confluence of approximately 70%. Resveratrol was dissolved in DMSO at a concentration of 100 mM and stored in aliquots at −20°C in a light-proof container. Resveratrol was used at a final concentration of 50 μM in growth media for 48 h. Leptomycin B (in DMSO) was used at a final concentration of 10 nM in growth media for 3 h. At the end of a treatment period, cells were washed with PBS, scraped off, collected by centrifugation and either directly used or flash frozen and stored at −80°C.

### Animals, Dietary Supplementation and Tissue Collection

All animal experiments were conducted in compliance with the Institutional Animal Care and Use Committee (IACUC) guidelines. Sexually-mature male Sprague-Dawley rats (Charles River, Wilmington, MA, USA) were housed in groups of three rats per cage under a strict 12 h light-dark cycle. The experimental animal group (*n* = 11) received dietary resveratrol mixed into a purified phytoestrogen-free AIN-76A basis diet (Harlan Teklad, Madison, WI, USA) provided *ad libitum* together with tap water for 28 consecutive days. Animals in the control group (*n* = 11) were fed the same dietary mixture without resveratrol. Animal weight and food consumption were recorded daily. On experimental day 29, animals were sacrificed by decapitation; whole trunk blood collected and allowed to clot for 2 h at room temperature. Serum was then separated by centrifugation (1800 g, 10 min) and stored frozen at −80°C until assayed. For gene expression and western blot analysis, rat brains were cut longitudinally into equal halves, flash frozen on dry ice and stored at −80°C.

### Determination of Serum Resveratrol Levels

Samples were hydrolyzed with beta-glucuronidase and extracted with ethyl acetate. The extracts were reconstituted in 5 mM ammonium acetate, pH 8.1: Methanol (50:50) and washed with hexane. The internal standard used was bis-(4-hydroxyphenyl)-methane. Due to concerns about the stability of resveratrol, the analysis preparation was performed in reduced room light and the extract evaporation used nitrogen instead of air. The extracts were analyzed by Liquid Chromatography–Mass Spectrometry using negative ion electrospray ionization (University of Utah, Center for Human Toxicology, Salt Lake City, UT, USA). The lower resolution level was set at10 ng/ml.

### Western Blot Analysis of Human Cells and Rat Brain Tissue

Rat brain halves or cell pellets were thawed on ice, pre-homogenized with sterile pestle and tubes (Kimble-Kontes, Vineland, NJ, USA). 30 μl of a pre-homogenate were mixed with 300 μl of radio immuno precipitation assay (RIPA) buffer for whole cell lysis [0.05 M Tris–HCl, pH 8.0, 1% NP-40, 0.5% deoxycholate, 0.1% SDS, protease inhibitor cocktail 1:500 (Sigma, Saint Louis, MO, USA)] and treated with an electric homogenizer followed by two blade rinses with RIPA buffer (100 μl each). Lysates were maintained under constant agitation (orbital shaker) for another 2 h at 4°C before centrifugation (20,000 g, 20 min at 4°C). Supernatants were assayed for protein concentration and stored in aliquots at −80°C. Upon thawing, samples were mixed with 2 × SDS-PAGE loading dye plus 100 mM DTT and heated to 95°C for 5 min for denaturation. Protein electrophoresis was carried out at equal protein loading (5 mg/ml) on 4–15% denaturing SDS-polyacrylamide gradient gels. Proteins were transferred onto nitrocellulose in a wet chamber (125 mA/gel, 90 min). Membranes were cut according to protein standards and probed 1:500 with either one of the following primary antibodies: polyclonal rabbit anti-human SIRT1 (Bethyl Laboratories, Montgomery, TX, USA); polyclonal rabbit anti-actin (H-300; Santa Cruz Biotechnology, Santa Cruz, CA, USA). For visualization, an appropriate horseradish peroxidase-coupled secondary antibody and color reaction were used according to the manufacturer (Thermo Fisher Scientific, Rockford, IL, USA). Results were recorded on a western-blot imaging system and exported into Adobe Photoshop CS6 (Adobe Systems Incorporated, Mountain View, CA, USA).

### Quantitative PCR (QPCR) on Human Cells and Rat Brain

For the *in vitro* determination of resveratrol-dependent changes in *sirt1* and *gpr50* expression (n ≥ 40), HEK-293 cells were grown and treated on multi-well dishes (6-well) and subsequently collected as described above. Total RNA was prepared separately from each well using the RNeasy RNA-isolation system in combination with QIA shredder columns according to the manufacturer’s specifications (Qiagen, Valencia, CA, USA). To determine the effect of dietary resveratrol on the expression of *sirt1* and *gpr50* in the brain, rat brain halves were pulverized with pestle, mortar and liquid nitrogen. Total RNA was prepared from 30 mg tissue samples representing the entire brain as delineated above. RNA concentration and integrity were determined with standard spectrophotometry and agarose gel-electrophoresis. Reverse transcription RT-PCR was performed using the Superscript III First-Strand Synthesis System for RT-PCR (Invitrogen/Life Technologies, Carlsbad, CA, USA). QPCR was carried out with the Power SYBR Green PCR Master Mix (Applied Biosystems, Foster City, CA, USA). DNA Primers were designed using the Integrated DNA Technologies PrimerQuest tool (IDT, Coralville, IA, USA). DNA primers used with human cells: *sirt1* forward: 5′-CTG TAG ACT TCC CAG ATC TTC CAG-3′; *sirt1* reverse: 5′-GTG ACA GAG AGA TGG CTG GAA TTG -3′; *β-actin* forward: 5′-CAG CCA TGT ACG TTG CTA TCC AGG-3′; *β-actin* reverse: 5′-AGG TCC AGA CGC AGG ATG GCA TG -3′; *gpr50* forward: 5′-CTT TGA TGC TGC ATG CCA TGT CCA-3′; *gpr50* reverse: 5′-TGT GGC AGA TGT AGC AGT AAC GGT-3′. DNA primers used with rat brain tissue: *sirt1* forward: 5′-ACA ACC TCC TGT TGG CTG ATG AGA -3′; *sirt1* reverse: 5′-AGA ATT GTT CGA GGA TCG GTG CCA-3′; *β-actin* forward: 5′-TGA GAG GGA AAT CGT GCG TGA CAT-3′; *β-actin* reverse: 5′-ACC GCT CAT TGC CGA TAG TGA TGA-3′; *gpr50* forward: 5′-AAA GGA AAT GGC AGG CAA GAT CCC-3′; *gpr50* reverse: 5′-ATG AAC AGG ATA GGG TGC CGC ATA-3′.

### ChIP of SIRT1 DNA Targets

For each experiment HEK-293 cells from 10 fully confluent 100 mm^2^ culture dishes were grown to 90–100% confluence. The EZ ChIP Kit (Upstate Technologies/EMD Millipore, Temecula, CA, USA) was used according to the manufacturer’s specifications for the generation and purification of SIRT1 specific genomic DNA. Briefly, after generating DNA/protein complexes through formaldehyde treatment of the cells, DNA was sheared by sonication of the cell lysate on wet ice into approximately 200–1000 base pair (bp) fragments (5 × 10 s pulses, 30% maximum power, 2 mm tip; Cell Disruptor, Heat-Systems Ultrasonics, Plainview, NY, USA). SIRT1/DNA complexes were immunoprecipitated with either 10 μg of a monoclonal (MC; Clone 2G1/F7, Upstate Technologies/EMD Millipore, Temecula, CA, USA) or a polyclonal antibody with human SIRT1 specificity (PC; Bethyl Laboratories, Montgomery, TX, USA). Standard rabbit Immunoglobulin G antibody (IgG) was used to generate the negative controls and a polyclonal rabbit antibody specific to acetylated histone H3 (AcH3) served to obtain positive controls (Upstate Technologies/EMD Millipore, Temecula, CA, USA). A sample of the initially cross-linked protein/DNA complexes was saved as input (IP).

### Cloning of SIRT1-Specific DNA Targets

Aliquots of 5 μl of the purified DNA solution obtained with monoclonal anti-SIRT1 antibody were treated with T4 DNA polymerase and dNTPs (Roche Applied Science, Mannheim, Germany) for 15 min at 16°C and subsequently heat inactivated at 75°C for 10 min. According to the manufacturer’s instructions, EcoRI adaptor oligonucleotides were ligated to the DNA pieces and excess adaptors subsequently removed with Sephacryl S-400 spin columns (Universal Riboclone cDNA Synthesis System, Promega, Madison, WI, USA). Purified DNA was amplified by standard PCR, using primer matching the EcoRI adaptor sequence (forward and reverse primer: AATTCCGTTGCTGTCG). Amplified DNA was briefly separated on 1.5% agarose gels from which a fragment range between approximately 200 bp–500bp was extracted (Qiagen, Valencia, CA, USA). Purified DNA was ligated into the pGEM-T Easy vector system and transformed into *E. coli* JM109 (Promega, Madison, WI, USA).

### Identification and Analysis of SIRT-Specific DNA Targets

Plasmid DNA from positive transformants (beta-galactosidase negative) was treated with EcoRI restriction endonuclease and analyzed by standard agarose gels electrophoresis (1.5%) to confirm presence of an insert. Confirmed clones were selected for DNA sequencing using standard T7 sequencing primer (T7 RNA polymerase promoter). DNA sequences were analyzed with the “Basic Local Alignment Search Tool” (BLAST; www.ncbi.nlm.nih.gov/blast/).

### Verification of SIRT1 Binding to the GPR50 Locus

GPR50-specific DNA-primers (forward: ACTACAGTAGGGTCAGGAAGGTCA; reverse: TAAACCCACCACTGGCCACATCAA; IDT, Coralville, IA, USA) were used together with a high-sensitivity PCR protocol (Soriano et al., 1991) on unamplified ChIP DNA obtained with both anti-SIRT1 antibodies. Gene-specific amplicons and proper controls were analyzed by analytical agarose gel electrophoresis (1.5%).

### Determination of Melatonin-Dependent Phospholipase C (PLC) Activity with ELISA

SH-SY5Y cells were grown to 70% density on 24-well plates before treatment. For a knockdown of SIRT1, cells were transfected with morpholino oligonucleotides (Gene Tools LLC, Philomath, OR, USA) for 4 h in serum-free medium with Lipofectamine 2000 (Invitrogen/Life Technologies, Carlsbad, CA, USA). At the same time, all non-transfected cells were treated with same serum-free medium and the transfection reagent without oligonucleotides to control for methodology-related variability. Anti-human SIRT1 and oligo-N random oligonucleotide control morpholinos (CMO) were applied at 100 pmol. Resveratrol was used for 48 h at 50 μM; melatonin was used for 48 h at 100 nM; luzindole was used for 48 h at 100 μM; the absolute control was treated with the solvent (DMSO) alone. Cells were washed twice with PBS, scraped off their well, collected by centrifugation and re-suspended in 130 μl PLC reaction buffer (Invitrogen/Life Technologies, Carlsbad, CA, USA) containing a protease inhibitor cocktail 1:500 (Sigma-Aldrich, St. Louis, MO, USA). Cells were lysed on ice by brief sonication for 3 s at maximum output control setting 3 (Cell Disruptor, Heat-Systems Ultrasonics, Plainview, NY, USA). Cell lysates were spun at 14,000 g for 5 min and 100 μl of each supernatant transferred to a pre-chilled 96-well microtiter plate. PLC activity was determined using the EnzChek Direct Phospholipase C Assay Kit according to the manufacturer’s specifications (Invitrogen/Life Technologies, Carlsbad, CA, USA). Fluorescence was measured with a standard ELISA reader at 2, 4, 18, 24, and 48 h (485/20, 528/20). The presented values were determined at 18 h and are representative for the entire experiment. Data are presented as the mean ± SEM. *p* < 0.05 by Student’s t test(s) were used for comparison with control (*); *n* = 12.

## Results

### SIRT1 Associates with the Gene Encoding the Orphan Receptor GPR50

**Figure 1:**
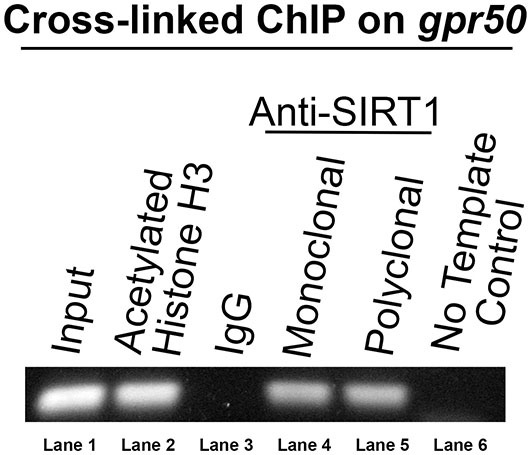
**Human *gpr50* is a SIRT1 DNA target.** DNA gel electrophoresis is shown of PCR amplicon on unamplified ChIP DNA with DNA primers matching genomic gpr50 at its most 3′exon and downstream non-coding region. The size of this amplicon is about 300 bp. Standard Taq-PCR was performed on the samples indicated. Two independent SIRT1 antibodies were used (lanes 4 and 5).

To identify specific genes that are transcriptionally regulated by SIRT1, we subjected HEK-293 cells to a ChIP assay with two unrelated anti-SIRT1 antibodies. Sequencing of the immunoprecipitated DNA identified a 300 bp genomic sequence overlapping most of the 3′ exon of *gpr50* and its downstream, non-coding region as a SIRT1 DNA target. To verify the presence of SIRT1 on this target locus, we used unamplified ChIP DNA in a PCR reaction with DNA primers matching the 3′ region of genomic *gpr50* (Figure 1). Both SIRT1-specific antibodies yielded identical results, thus supporting our initial findings.

### Resveratrol Treatment Increases *gpr50* Expression through SIRT1 in HEK-293 Cells

**Figure 2:**
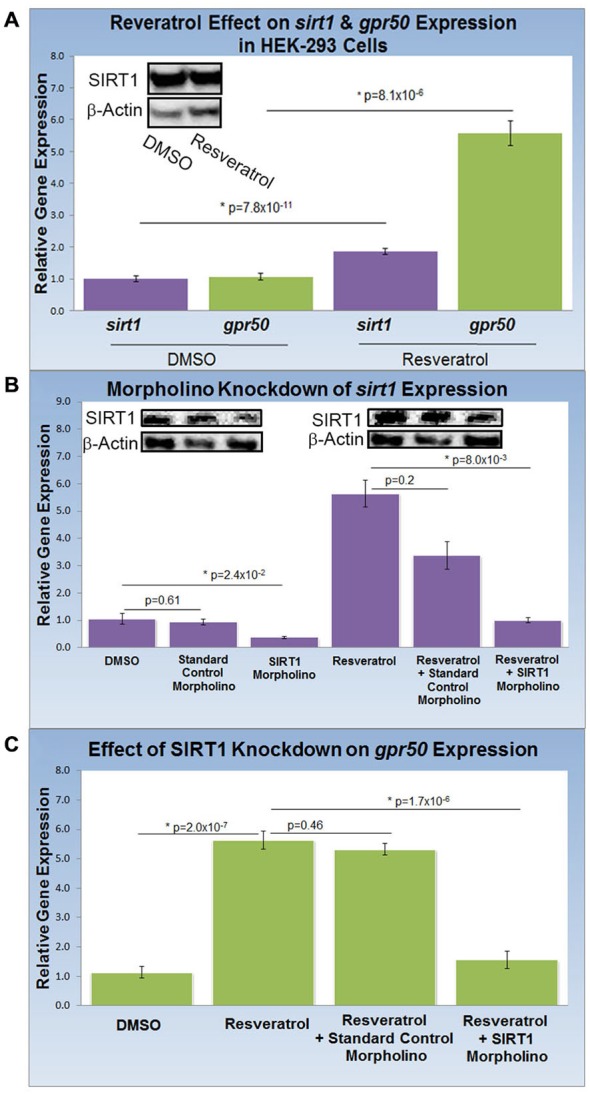
**Knockdown of SIRT1 reverses resveratrol effects on *gpr50* expression in HEK-293 cells. (A)** Quantitative PCR (QPCR) analysis of resveratrol-induced (50 μM; 48 h) transcriptional changes compared to vehicle only (DMSO). Resveratrol treatment induces significant transcriptional increases in *sirt1* (1.9 fold) and *gpr50* (5.6 fold). Western-blot analysis supports resveratrol-dependent stimulation of SIRT1 protein levels (inset) **(B)** QPCR and western blot validation of *sirt1* morpholino knockdown from basal levels (columns1–3) and after resveratrol induction (columns 4–6). Values are means ± SD. *Indicates statistical significance of annotated *p*; *n* = 20. **(C)** Morpholino oligonucleotide knockdown of *sirt1* reverses resveratrol-induced *gpr50* transcriptional increases [5.6 fold→ 1.6 fold (columns 2 and 4)]. The inclusion of a standard control morpholino mix has no significant effect (column 3).

To study the effect of resveratrol on the expression of *sirt1* and its gene target, *gpr50* (*n* = 20 for all experiments), we compared the transcription activity of both genes with and without resveratrol (Figure 2A). Resveratrol treatment (50 μM) of HEK-293 cells for 48 h caused a significant transcriptional increase of *sirt1* of 1.9 fold (*p* = 8.1 × 10^−6^) along with an average increase of *gpr50* transcription of 5.6 fold (*p* = 7.8 × 10^−11^) plus a notable increase in SIRT1 protein levels (Figure 2A). To demonstrate SIRT1 dependence of the observed *gpr50* transcriptional increase in HEK-293 cells, we administered previously tested (Torres et al., 2011) morpholino oligonucleotides (morpholinos) with or without resveratrol. Application of a *sirt1*-specific morpholino reduced native- as well as resveratrol-enhanced *sirt1* transcription significantly by about 60% (*p* = 2.4 × 10^−2^) and 70% (*p* = 8 × 10^−3^) respectively, whereas the application of a standard control morpholino mix showed no considerable effect (*p* = 0.61). These findings are in agreement with SIRT1 protein levels as shown in (Figure 2B). Using HEK-293 cells, we further found that application of *sirt1*-specific morpholino significantly reduced resveratrol-dependent *gpr50* transcript levels from 5.6-fold to about 1.6 fold (*p* = 1.7 × 10^−6^), whereas cellular transfection with CMO did not exert any significant effect at all (*p* = 0.46; Figure 2C).

### Dietary Resveratrol Increases *gpr50* Expression in Rat Brain

**Figure 3:**
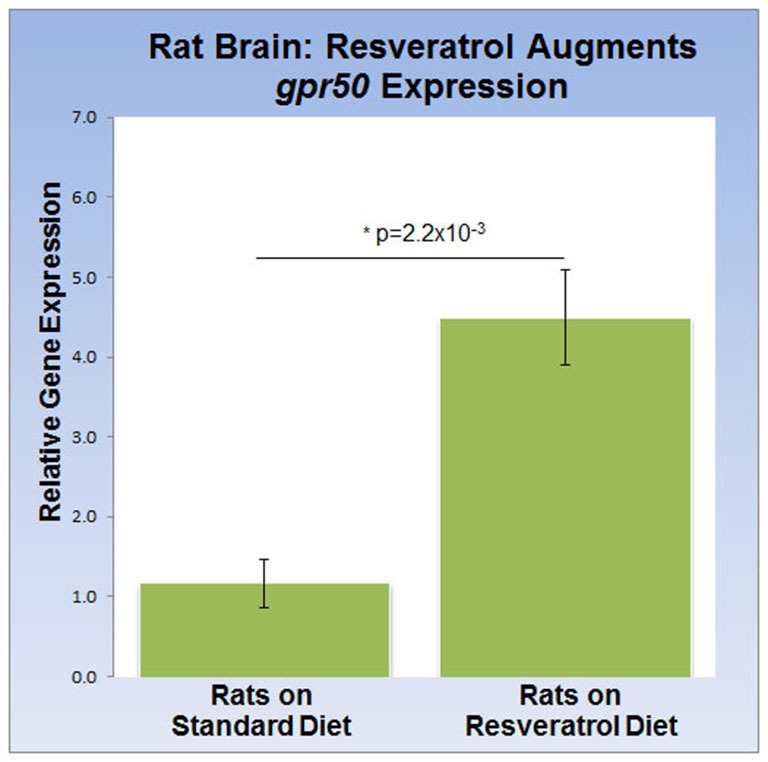
**Dietary resveratrol stimulates *gpr50* transcription in rat brain.** Analysis of whole brain extracts of animals fed resveratrol (1 g/kg chow; 4 weeks) shows a significant increase in *gpr50* transcription of 4.5 fold. Values are means ± SEM. *indicates statistical significance of annotated *p*; *n* = 11 animals per group.

To address the effects of dietary resveratrol on the expression of *sirt1* and *gpr50* in the mammalian brain, we analyzed whole brain tissue from rats exposed to a 4-week resveratrol diet regiment. Average serum levels of resveratrol were found to be 349.2 ± 97 ng/ml in the experimental group, but predictably absent in the control group. Whereas transcription of *sirt1* (QPCR) and protein levels (western-blot) were indistinguishable between experimental and control rats (data not shown), we found that *gpr50* transcription was significantly up-regulated by 4.5 fold (*p* = 2.2 × 10^−3^) in the resveratrol-fed group (Figure 3).

### PLC Activity and *gpr50* Transcription are Reversibly Linked in Neurons with a Dopamine Phenotype

**Figure 4:**
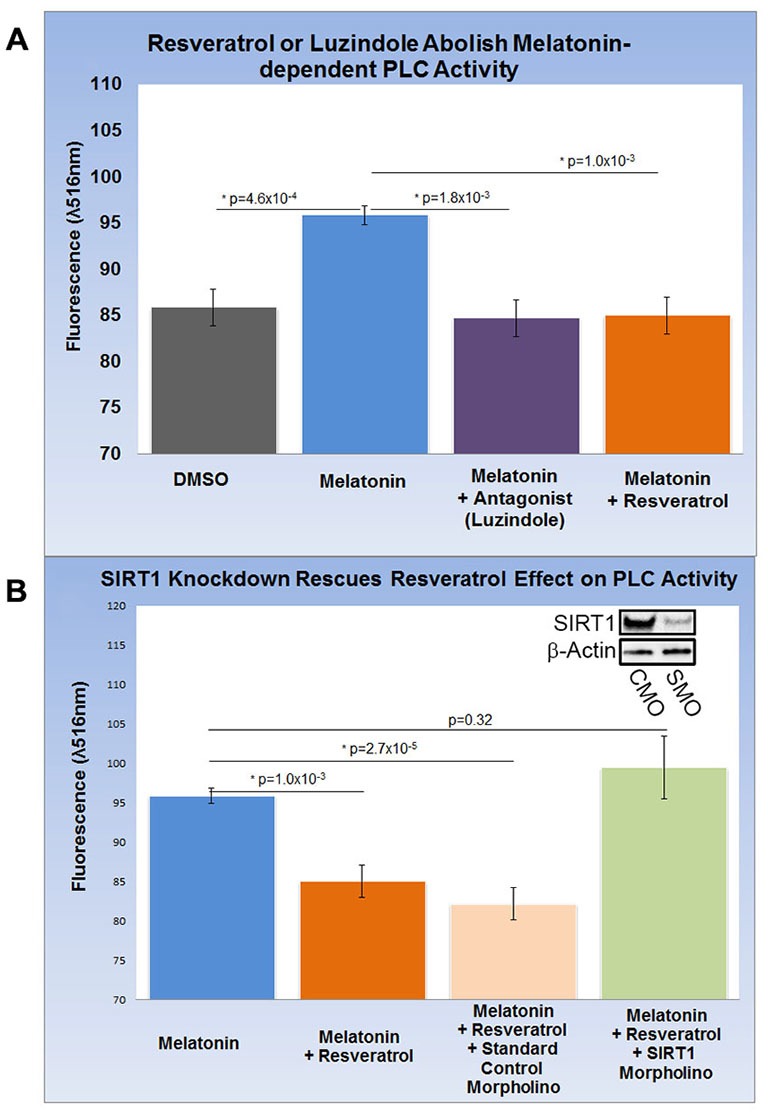
**Increased *gpr50* transcription decreases PLC activity in SH-SY5Y cells with a matured dopamine neuronal phenotype.** Two ELISA fluorescence readout (λ516 nm) experiments with relevant groups were performed. **(A)** Whereas melatonin treatment increases PLC activity from baseline by about 10%, luzindole (selective MT1, MT2 antagonist) as well as resveratrol completely reverses this effect. **(B)** Melatonin-mediated increases in PLC activity are reversed by resveratrol while addition of standard control morpholino mix (CMO) has no significant effect. The resveratrol-effect on PLC activity is reversed by the addition of a SIRT1-specific morpholino (SMO) even in the presence of resveratrol. SIRT1 protein levels (inset) are sensitive to SMO but not CMO. Data are presented as the mean ± SEM. *p* < 0.05 by Student’s *t* test for comparison with control (*); *n* = 12 for all groups.

HEK-293 cells subjected to melatonin treatment did not yield reproducible changes in PLC activity (data not shown) and were therefore excluded from this assay. Encouraged by our findings in rat brain, we differentiated SH-SY5Y human neuroblastoma cells into functionally-matured DA neuronal phenotypes and found that melatonin treatment increased PLC activity modestly but significantly by more than 10% (*p* = 4.6 × 10^−4^). This increase was entirely abolished by luzindole (*p* = 1.8 × 10^−3^) pointing to the specific involvement of MT1 and MT2 (Figure 4A). Next, we determined the effect of resveratrol on melatonin-dependent PLC signaling and found that resveratrol (*p* = 1.0 × 10^−3^) had a similar effect on PLC activity as luzindole (Figures 4A,B). To probe for the role of SIRT1 in this resveratrol-dependent effect, we applied *sirt1*-specific (SMO) and standard CMO and confirmed their respective effect on SIRT protein levels with western blots (Figure 4B). While melatonin-dependent PLC activities were significantly reduced in the resveratrol (*p* = 1.0 × 10^−3^) and resveratrol + CMO (*p* = 2.7 × 10^−5^) groups, morpholino-dependent SIRT1 knockdown completely rescued melatonin-dependent PLC activity close to original levels (*p* = 0.32). These results suggest the involvement of SIRT1 in melatonin-induced changes of PLC activity in SH-SY5Y cells.

## Discussion

SIRT1 is a protein deacetylase widely recognized for its involvement in double-stranded DNA repair, chromosomal stability and chronological aging in vertebrate and invertebrate species (Blander and Guarente, 2004; Michan and Sinclair, 2007). SIRT1 also has gained considerable attention for its role in regulating energy metabolism in response to dietary changes, namely caloric restriction (Michan and Sinclair, 2007). In mammals, SIRT1 is ubiquitously localized to the entire breath of the central nervous system, including discrete anatomical clusters generating circadian rhythms such as neurons of the anterior hypothalamus (Zakhary et al., 2010). Among the signaling cues that activate SIRT1-dependent signaling pathways, at least in laboratory animals, is the regular consumption of resveratrol (Baur and Sinclair, 2006; Torres et al., 2011). These findings suggest that the brain is particularly sensitive to bio-molecules that mimic physiological adaptations typical of energy restriction, and also highlight the importance of SIRT1 in regulating key transcriptional steps in circadian oscillation. Indeed, metazoan circadian rhythms are tightly coupled to glucose metabolism and bioenergetics, including metabolic pathways associated with the daily or seasonal activity of feeding and fasting cycles (Mattson et al., 2014; Tasselli and Chua, 2015).

Our present findings provide further evidence on the importance of SIRT1 in linking circadian rhythm components and metabolic pathways both *in vitro* and *in vivo* experimental conditions. Specifically, we show that SIRT1 directly associates with the gene encoding *gpr50* leading to its up-regulation following the consumption of resveratrol during the rat’s subjective night phase of the light-dark cycle. Although the exact mechanism of this association needs yet to be described, SIRT1 association with transcriptional complexes facilitating gene activation has been previously documented. For instance, SIRT1 appears to interact with, and activate, transcription of the peroxisome proliferator activated receptor-γ co-activator-1α (PGC-1α) gene which controls overall metabolic function (Amat et al., 2009). Along this theme, SIRT1 is known to mediate transcriptional activation of neuroprotective pathways preventing Huntington’s disease (Jeong et al., 2011). Another line of evidence strengthening the connection between circadian regulation and our current findings comes from work conducted in GPR50 knockout mice. While GPR50 knockout animals do not exhibit significant alterations in overall circadian periodicity, their nocturnal and diurnal activity levels and metabolic rates are significantly increased. A closer look at the knockout reporter gene expression in place of *gpr50* reveals a close link to the cellular energy status (Ivanova et al., 2008) which falls in line with the metabolic paradigm underlying the above resveratrol-SIRT1 association. Finally lack of *gpr50* expression in the hypothalamus, fails to respond to leptin and thyrotropin-releasing hormone signals (Bechtold et al., 2012); hormone signals that are closely intertwined in circadian rhythmicity and metabolic regulation, respectively.

**Figure 5:**
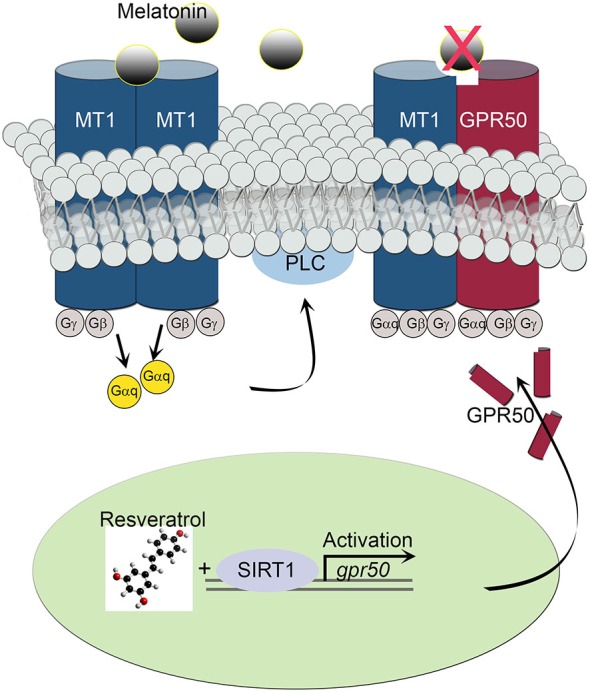
**Schematic diagram depicts the effect of MT1/GPR50 heterodimer formation on PLC signaling events.** Whereas MT1/MT1 G protein-coupled transmembrane homodimers are able to transduce photoperiodic information via the activated α subunit of a trimeric (αβγ) G protein Gαq to PLC, MT1/GPR50 heterodimers are unable to bind melatonin and remaining inactive. The balance of active vs. inactive transmembrane melatonin receptors is dependent on the transcriptional activity of *gpr50* governed by SIRT1 and resveratrol stimulus.

The transcriptional effect of the melatonin-related receptor gene *gpr50* is also seen in HEK293 human cells exposed to doses of 50 μM resveratrol. These results, together with the observed downstream effect on PLC activity, imply a functional relationship between SIRT1 and the melatonin signaling machinery at the cellular as well at the organismal level. Furthermore, the above results highlight the importance of a resveratrol diet, mimicking calorie restriction, in coupling SIRT1 with photoperiodic information, with the potential to affect light-dark and sleep-wake cycles. In line with this notion, resveratrol treatment obstructed the activity of intracellular PLC often initiated by melatonin’s diffused signals in SH-SY5Y human neuroblastoma cells. Both, homodimeric and heterodimeric MT1/MT2 G protein-coupled receptor-agonist complexes, activate PLC via the Gq protein to stimulate the phosphoinositol second-messenger cascade, which in turn, triggers the activation of protein kinase C and subsequent steps (Ayoub et al., 2004; Jockers et al., 2008). For instance, whereas MT2/GPR50 heterodimers are active participants in melatonin signaling transduction, MT1/GPR50 complexes appear to be inert, shifting overall PLC signaling to a less active state in response to SIRT1-dependent transcriptional regulation (Figure 5). This sequence of intracellular events is thought to enable melatonin signals to synchronize synaptic activity, circuit information and network connectivity to the daily light-dark cycle (Pandi-Perumal et al., 2008; Baba et al., 2013).

While light is the dominant timing cue for melatonin circadian rhythm and for the entrainment of the SCN oscillator in the anterior hypothalamus, our results suggest that resveratrol consumption can also affect the activity of transmembrane receptor-dependent melatonin signaling via SIRT1 activity. As noted in the introductory paragraphs, SIRT1 is involved in the transcriptional activation of circadian rhythms, particularly diurnal rhythms associated with food intake and glucose metabolism (Mattson et al., 2014; Luna et al., 2015). These observations clearly suggest that food consumption can indirectly alter the melatonin-related receptor GPR50 and thus likely shape unique network properties of the circadian clock in the hypothalamus and/or peripheral tissues where the circadian clock is cell-autonomous (Bechtold et al., 2012). In this context, the GPR50 is implicated in adaptive thermogenesis and torpor in rodents and in the pathophysiology of bipolar disorder and major depression in women; brain disorders characterized by cognitive and emotional dysregulation (Grünewald et al., 2012). These observations suggest that specific components of foodstuffs can profoundly alter brain function, supporting the notion that the human brain may be particularly sensitive to changes in caloric restriction and circadian timing of meals (Mattson et al., 2014).

In conclusion, our results support and extend the possibility that a number of biomolecules and druggable brain targets may be used to treat pathologies of the ailing brain. In the near future, it is conceivable that brain disorders may require a broader approach in which nutritional foodstuffs are essential in order to provide better health outcomes, functioning and quality of life.

## Conflict of Interest Statement

The authors declare that the research was conducted in the absence of any commercial or financial relationships that could be construed as a potential conflict of interest.
